# Molecular gatekeepers: eukaryotic translation factors decoding plant–virus dynamics for resistance engineering

**DOI:** 10.1007/s44154-025-00273-2

**Published:** 2026-01-27

**Authors:** Pankhuri Singhal, Shubham Saini, Oshin Saini, Ankit Bishnoi, Rashmi E.R., Bharat Raj Meena, Jitender Singh, Kalenahalli Yogendra

**Affiliations:** 1 Division of Plant Pathology, Advanced Centre for Plant Virology, ICAR-Indian Agricultural Research Institute, New Delhi, 110012 India; 2 Department of Plant Pathology, CCS Haryana Agricultural University, Hisar, Haryana 125004 India; 3 Department of Plant Pathology, Punjab Agricultural University, Ludhiana, Punjab 141004 India; 4 ICAR-Central Institute of Temperate Horticulture, Regional Station, Mukteshwar, Uttarakhand 263138 India; 5 Division of Plant Quarantine, ICAR-National Bureau of Plant Genetic Resources, New Delhi, 110012 India; 6 Department of Microbiology, Chaudhary Charan Singh University, Meerut, 250004 India; 7 International Crops Research Institute for the Semi-Arid Tropics, Hyderabad, 502324 India

**Keywords:** Eukaryotic translation initiation factors, Virus translation, Movement, Replication, Eukaryotic translation elongation factors

## Abstract

Plant viruses are among the most significant biotic stressors, posing a severe threat to crop productivity and global food security. Their success largely depends on the exploitation of host eukaryotic translation factors (eTFs), including initiation factors (eIFs) and elongation factors (eEFs), which act as molecular gatekeepers of the viral life cycle. Key members such as eIF4E, eIF(iso)4E, eIF4G, eEF1A, and eEF1B have been identified as susceptibility factors that mediate viral translation, replication, and systemic movement. Viruses have co-evolved specialized proteins and RNA elements, including VPg and IRES structures, to hijack these host factors and circumvent plant defense barriers. This review synthesizes current understanding of the mechanistic roles of eTFs in virus–host dynamics and highlights strategies to mitigate viral stress. Approaches such as natural allele mining, induced mutagenesis, TILLING/EcoTILLING, RNA interference, and precise genome editing with CRISPR/Cas systems are explored as practical tools for reducing susceptibility. Targeted manipulation of eTFs offers a promising avenue to reprogram plants for resistance while maintaining essential cellular functions. By integrating molecular biology with applied strategies, we propose an eTF-centered framework for resistance breeding within a broader stress biology perspective. Future research combining functional genomics, synthetic biology, and breeding innovation will be pivotal in delivering broad-spectrum, durable, and environmentally sustainable resistance to plant viral stress.

## Introduction

Plant viruses are significant pathogens that pose a substantial threat to global agriculture. They are accountable for almost half of all plant diseases, underscoring their pervasive impact on crop health (Hilaire et al. 2022). Annually, plant virus diseases account for yield reductions estimated at approximately $30 billion worldwide, affecting both the quantity and quality of agricultural produce, jeopardizing food security (Tatineni and Hein 2023). The infection cycle of plant viruses encompasses several critical stages: initial entry into host cells, uncoating of the viral genome, translation of proteins, replication of RNA/DNA, assembly of new virions, and movement within the plant, both cell-to-cell and systemically. Given their limited genomic capacity, plant viruses rely extensively on host cellular machinery to facilitate these processes. Among the host factors co-opted by viruses, eukaryotic translation initiation factors (eIFs) and eukaryotic translation elongation factors (eEFs) play pivotal roles. These factors are not only essential for the translation of viral RNAs but also contribute to viral replication and movement within the host (Sanfaçon 2015; Zlobin and Taranov 2023).

The host factors exploited by viruses for completion of the infection cycle are termed susceptibility factors or the pro-viral factors, as their absence or inaccessibility renders the host resistant to infection (Diaz‐Pendon et al. 2004). Thus, a resistant phenotype is produced when a specific host factor necessary for the virus cycle is mutated, weakening the plant-virus interaction. Being inherited recessively, it is known as recessive resistance, which is considered to be more durable and non-specific as compared to dominant resistance conferred by resistant genes (Zlobin and Taranov 2023).

Understanding the intricate interactions between plant translation factors and viruses, and elucidating the molecular mechanisms, is crucial to devising targeted interventions to disrupt the viral lifecycle. This knowledge paves the way for innovative approaches in crop protection, including the engineering of eIF variants that confer broad-spectrum resistance without compromising essential plant functions.

**Fig. 1:**
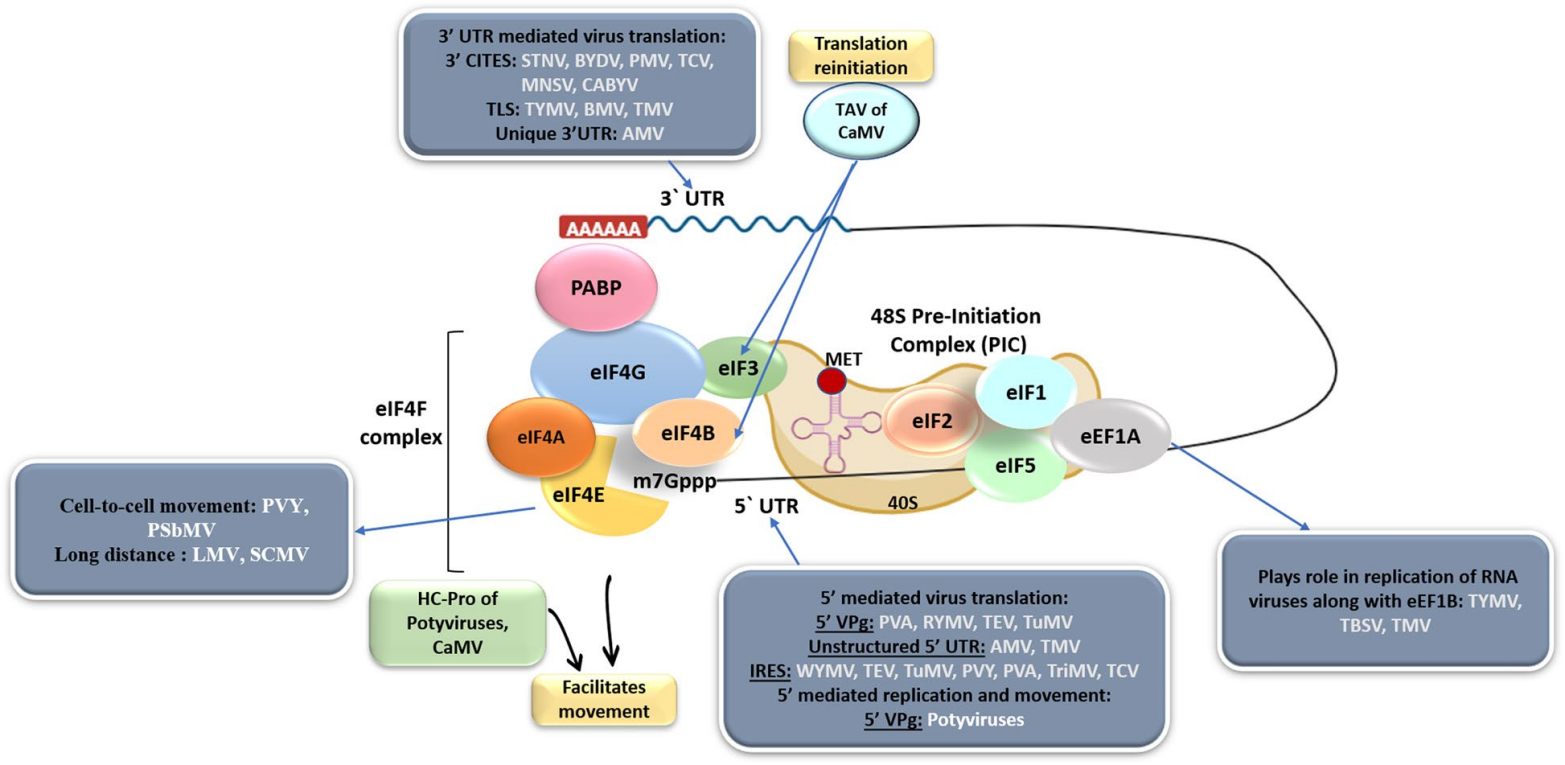
Schematic representation of the role of eukaryotic translation initiation and elongation factors (eIFs and eEFs) in eukaryotic translation and their exploitation for plant virus infection processes. The figure illustrates different plant viruses (STNV, BYDV, PMV, TCV, MNSV, CABYV, TYMV, BMV, TMV, AMV, CaMV, PVY, PSbMV, LMV, SCMV, PVA, RYMV, TEV, TuMV, WYMV, TriMV, TBSV) along with molecular components involved in translation regulation (3′UTR, 3′CITEs, TLS, TAV, PABP, eIF, HC-Pro, VPg, IRLS-MET, PIC). satellite tobacco necrosis virus (STNV), barley yellow dwarf virus (BYDV), panicum mosaic virus (PMV), turnip crinkle virus (TCV), melon necrotic spot virus (MNSV), cucurbit aphid-borne yellows virus (CABYV), turnip yellow mosaic virus (TYMV), brome mosaic virus (BMV), tobacco mosaic virus (TMV), alfalfa mosaic virus (AMV), cauliflower mosaic virus (CaMV), potato virus Y (PVY), pea seed-borne mosaic virus (PSbMV), lettuce mosaic virus (LMV), sugarcane mosaic virus (SCMV), potato virus A (PVA), rice yellow mottle virus (RYMV), tobacco etch virus (TEV), turnip mosaic virus (TuMV), wheat yellow mosaic virus (WYMV), Triticum mosaic virus (TriMV), and tomato bushy stunt virus (TBSV), the 3′ untranslated region (3′UTR), 3′ cap-independent translation element(s) (3′CITEs), and the transfer RNA-like structure (TLS) play crucial roles in viral translation. Similarly, transactivation-viral protein (TAV), poly(A)-binding protein (PABP), eukaryotic initiation factor (eIF), helper component-proteinase (HC-Pro), viral protein genome-linked (VPg), and pre-initiation complex (PIC)

Translation initiation and elongation factors are increasingly recognized not only as essential components of the host protein synthesis machinery but also as multifunctional nodes exploited by diverse plant viruses (Fig. 1). Their ability to perform overlapping, context-dependent roles—ranging from mRNA recognition and ribosome recruitment to regulatory interactions with viral proteins such as VPg—highlights their dual significance in normal plant physiology and in disease susceptibility. This multifunctionality raises a key scientific question that anchors the present review: how can the inherent versatility of translation factors be leveraged to develop broad-spectrum and durable resistance against plant viruses? By integrating structural insights, omics-driven discoveries, and advances in functional genomics, we aim to synthesize current knowledge around this focal point and outline strategies to translate mechanistic understanding into resistance breeding and biotechnological applications.

## Role of eIFs in host plants and the virus infection cycle

### Role in eukaryotic translation

Eukaryotic translation is a complex, cyclical process comprising four phases: initiation, elongation, termination, and recycling. Initiation begins with the assembly of the 43S preinitiation complex (PIC), which includes the 40S ribosomal subunit, the eIF2–GTP–Met-tRNAiMet ternary complex (eiF2TC), eIF3, eIF1, eEF1A, and eIF5. This complex binds to the mRNA’s 5′ cap with the help of the eIF4F complex, consisting of eIF4E, eIF4A, and eIF4G, along with eIF4B and eIF4H, which unwind the secondary structures in the 5′ untranslated region (5′ UTR) to facilitate ribosomal attachment (Gross et al. 2003; Volpon et al. 2006). The 43S complex then scans the mRNA from 5′ to 3′ until the initiation codon (typically AUG) is located, with eIF1 playing a critical role in ensuring fidelity by preventing incorrect codon recognition (Pestova and Kolupaeva 2002). Upon start codon recognition, the 48S complex is formed, and eIF5B promotes joining of the 60S ribosomal subunit to form the elongation-competent 80S ribosome. Elongation involves codon recognition, peptide bond formation, and ribosomal translocation, mediated by eEF1A (eukaryotic elongation factor 1 A or 1α) and eEF2 in a GTP hydrolysis-dependent manner, allowing aminoacylated tRNAs to deliver amino acids to the growing polypeptide chain. The eIF2α is part of the heterotrimeric eIF2 complex (α, β, γ subunits), which delivers the initiator Met-tRNAi to the 40S ribosomal subunit to form the ternary complex (eIF2–GTP–Met-tRNAi). The phosphorylation of eIF2α (at Ser51) by its kinase GCN2 in plants acts as a major checkpoint. When phosphorylated, eIF2α sequesters eIF2B (the guanine nucleotide exchange factor), preventing conversion of GDP to GTP, halting global protein synthesis. Termination is triggered when a stop codon (UAA, UGA, or UAG) is recognized by eukaryotic release factors (eRFs), leading to peptide release, subunit dissociation, and ribosome recycling with the assistance of ABCE1 and other factors (Rees et al. 2009). Recycling disassembles the post-termination complex into free ribosomal subunits, mRNA, and tRNA, facilitated by eIF3, eIF1, and eIF1A. Translation regulation, particularly during initiation, is critical, with stress-induced phosphorylation of eIF2α inhibiting GDP-GTP exchange, thereby preventing initiator tRNA delivery, a mechanism often exploited during viral infections to suppress host protein synthesis (Wek 2018).

### Other functions in plants

In addition to their primary role in translating cellular mRNAs, eIFs exhibit diverse biological functions. For example, eIF4E is predominantly localized in the cytoplasm of *Arabidopsis thaliana* cultured cells, but its nuclear-cytoplasmic partitioning appears to be regulated by the cell growth cycle (Bush et al. 2009). This dynamic localization suggests that eIF4E may be context-dependent in nuclear export processes. Similarly, eIFiso4E, displays an equal distribution between the nucleus and cytoplasm, further supporting the hypothesis that plant eIF4E and eIFiso4E could participate in nuclear export of specific mRNAs. The eIFiso4F complexes, comprising eIFiso4E and eIFiso4G, associate with microtubules, promoting their end-to-end annealing (Browning 2004). A direct interaction mediates the localization of eIFiso4F to microtubules between eIFiso4G and the microtubules. This interaction indicates a regulatory role in microtubule organization and stability and critical processes for cellular structure and intracellular transport. The eEF1A is reported to be involved in the regulation of nuclear export, stabilization, organization of the cytoskeleton and microtubules, and protein stability (Mateyak and Kinzy 2010). These activities are mediated through interactions with the proteasome and other cellular components, positioning eEF1A as a central player in maintaining cellular homeostasis. These non-canonical roles highlight the multifaceted nature of eIFs and their potential as targets for viral exploitation.

### Role of eIFs in the virus infection cycle

Viruses have evolved to exploit the non-canonical roles of eIFs and related factors to enhance their own lifecycle. By hijacking the nuclear export machinery, cytoskeletal regulation, and protein stability pathways mediated by eIFs, viruses can stabilize their proteins, optimize their replication processes, and facilitate intra- and intercellular movement.

#### Viral protein translation

After entering the host cell, the viral particles undergo disassembly to expose their RNA (acting as mRNA) for protein synthesis. Viruses manipulate host translation by targeting factors like eIF4E and eIF4F complex facilitating ribosome recruitment, scanning and elongation; prioritizing viral mRNA translation over host mRNA (Pestova and Kolupaeva 2002). The phosphorylation of eIF2α by GCN2 in plants has been implicated as essential for the initiation of mRNA translation in many viruses (Liu et al. 2020). For translation, interaction occurs at the molecular level between various genomic and extra-genomic (located at the 5′ and 3′ untranslated) regions of the virus with eIFs. For example, the potyviral VPg proteins interact with eIF4E or eIF(iso)4E to mediate the initiation of virus translation in a cap-independent manner. Similarly, viral internal ribosome entry sites (IRESs) and 3′ cap-independent translational enhancers (CITEs) act to recruit the host translation machinery due to the absence of a 5′ cap structure to initiate translation of viral RNA (Simon and Miller 2013). The details have been provided in the various subsections.

#### Replication

The translation factors like eEF1A, eEF1B, and eIF4A, etc., link with viral replication complexes (VRCs) mediating the replication of *Tobamoviruses*, *Tombusviruses*, and *Potyviruses* (Nishikiori et al. 2006; Thivierge et al. 2008; Hwang et al. 2013; Kovalev and Nagy 2014). The interaction between the eEF1A, 3′ t-RNA-like structure (TLS) of turnip yellow mosaic virus (TYMV) and RNA-dependent RNA polymerase (RdRp) is found to regulate the synthesis of the negative RNA strand (Matsuda et al. 2004). Similar interaction has been reported for tomato bushy stunt virus (TBSV) wherein eEF1A interacts with the 3′ cis-acting element, viral RdRp, enabling recruitment of viral RNAs in the VRC, enhancing (-) strand RNA synthesis (Li et al. 2009).

#### Cell-to-cell and long-distance movement

The eIF4E and its isoforms have been described to facilitate viral cell-to-cell (Arroyo 1996) and systemic movement (Rodríguez-Gómez et al. 2022) by binding to the coat protein (CP) of the viruses (Contreras-Paredes et al. 2013). This could be due to the eIF4G's binding affinity to the microtubules, which is the binding partner of eIF4E.

## Viral regions interacting with eukaryotic translation factors

Viral RNAs that lack a cap contain specific structures that enable them to interact with the Plant Translation Factors (PTFs), enabling cap-independent translation of viral proteins (if they did not, there would be no translation). These structures are most commonly located on the 5′ leader or the 3′ terminal sequences (Simon and Miller 2013; Sorokin et al. 2021). However, a few coding regions of the viral genome also aid in this interaction, as discussed below (Fig. 1).

### Unstructured 5′ untranslated regions (5' UTR)

Some viral mRNAs possess unstructured 5′ untranslated regions (5′ UTRs) to achieve efficient translation, often circumventing the need for canonical initiation factors. These unstructured 5′ UTRs are highly adaptive and give viruses a competitive advantage in host cells. Such as alfalfa mosaic virus (AMV), whose subgenomic RNA 4 contains a 36-nucleotide (nt), U-rich, unstructured 5′ UTR. This region interacts with the eIF4F, enabling efficient translation without a 5′ cap (Hann and Gehrke 1995). The tobacco mosaic virus (TMV) RNA features a 67 nt, A-rich omega leader sequence with single-stranded conformation acting as a translation enhancer for TMV mRNA (Gallie et al. 1987).

### 5′ Viral protein genome-linked (Vpg)

The VPg is a multifunctional protein covalently attached to the 5′ end of positive-sense single-stranded RNA (ssRNA) genomes of many plant viruses of family *Potyviridae*, *Secoviridae*, and genus *Sobemovirus* (Jiang and Laliberté 2011). VPg plays a pivotal role in RNA synthesis, translation, and potentially other stages of the viral infection cycle by serving as an m7G cap analogue of the mRNA (Ivanov et al. 2014; Choudhary and Suresh 2020). The essential role of VPg in potyvirus replication was confirmed through experiments where protease-mediated cleavage of VPg from viral RNA inhibited replication (Sanfaçon 2015). Mutations in VPg that reduce its binding to eIF4E/iso4E significantly impair viral replication and systemic infection (Saha and Mäkinen 2020). VPg forms a complex with eIF genes and other viral genomic regions, such as HC-Pro, forming the VPg-Pro-eIF4E/eIF(iso)4E complex, which localizes to subnuclear structures and cytoplasmic vesicles embedded in the ER, facilitating viral RNA movement (Beauchemin et al. 2007). The VPg facilitates the systemic movement of the tobacco etch virus (TEV) by binding to the eIF4E and CP (Contreras-Paredes et al. 2013).

It also mimics the 5′ cap structure of mRNA, enabling the viral genome to recruit host translation machinery for protein synthesis effectively (Wang and Krishnaswamy 2012). VPg’s mimicry of the mRNA cap structure allows it to outcompete cellular mRNAs for translation initiation, prioritizing viral genome translation (Khan et al. 2008), a strategy observed in potato virus A (PVA) and rice yellow mottle virus (RYMV) (Hébrard et al. 2010; Eskelin et al. 2011). Additionally, VPg’s binding is often stabilized by eIF4G/eIFiso4G, forming a complex critical for effective virus infections (Michon et al. 2006; Nicaise et al. 2007; Wittmann et al. 1997).

In recent years, novel approaches have greatly advanced our understanding of how viral genome-linked proteins (VPgs) manipulate the host translation machinery, although these insights remain underrepresented in the plant virology literature. High-resolution structural studies, including cryo-electron microscopy (cryo-EM) and molecular dynamics simulations, have begun to define the precise molecular surfaces mediating eIF4E–VPg interactions. For example, a single amino acid substitution within the α1–α2 loop of VPg was recently shown to enable papaya ringspot virus (PRSV) to overcome eIF4E-mediated resistance in watermelon by re-hijacking a different isoform, eIF(iso)4E, illustrating the structural plasticity that underlies host adaptation (Zhou et al. 2024b). Complementary biochemical work has demonstrated that casein kinase 2-mediated phosphorylation of eIFiso4E/eIFiso4G enhances VPg binding, and that poly(A)-binding protein (PABP) further stabilizes this interaction, providing mechanistic insights into how VPg can mimic the m7G cap and redirect host translation (Khan et al. 2024). Parallel to these structural insights, systems-level investigations are beginning to uncover how VPg-mediated hijacking influences host gene expression.

### Internal ribosome entry sites (IRES)

Internal ribosome entry sites (IRESs) are structural RNA motifs located at the 5′ of the viral RNA that offer another non-canonical translation mechanism found in many (+) ssRNA viruses. To synthesise viral proteins, IRESs have developed into specific folds that attract host ribosomes to initiate internal translation of the viral RNA, bypassing the need for a 5′ cap (Mailliot and Martin 2018). IRESs have been reported in many plant viruses like wheat yellow mosaic virus (WYMV), TEV, TuMV, potato virus Y (PVY), PVA, Triticum mosaic virus (TriMV), turnip crinkle virus (TCV), etc. (Geng et al. 2021; Jaramillo-Mesa et al. 2019; May et al. 2017; Zhang et al. 2015). The IRES interacts with various eIFs, such as 4G, 4 F, etc., to enhance the translation of viral RNAs (Gallie 2001). For example, TriMV 5′-UTR interacts with eIF4G or eIFiso4G and mediates translation initiation by requiring eIF4A helicase activity (Roberts et al. 2017).

### Cap-independent translation enhancers (CITES)

Some positive-strand RNA plant viruses have evolved specialized RNA elements referred to as cap-independent translation enhancers (3′ CITEs) located in their 3′ untranslated regions (3′ UTRs) that enhance viral RNA translation by mimicking the cap-dependent translation process (Geng et al. 2021; Sanfaçon 2015). CITEs interact with key translation initiation factors, particularly components of the eIF4F complex, including eIF4E and eIF4G, as well as ribosomal subunits (Du et al. 2017; Kraft et al. 2019). A hallmark of CITEs is their ability to establish long-distance RNA-RNA interactions, specifically kissing-loop interactions, between a 5′-proximal hairpin loop and the 3′-CITE, effectively bridging the two ends of the viral genome to facilitate translation (Gao et al. 2018). These interactions, often mediated by conserved sequence motifs (e.g., YGCCA/UGGCR), bring the two ends of the viral RNA into proximity, facilitating ribosome scanning and translation initiation. CITEs are categorized into several structural and functional types based on their mechanisms of translation enhancement and interactions with translation machinery.

#### Translation Enhancer Domain (TED)

The TED was the first identified CITE in the satellite tobacco necrosis virus (STNV) (Danthinne et al. 1993). It is characterized by a long stem-loop structure with multiple bulges, facilitating direct binding to eIF4F and the isoform to initiate translation, bypassing the 5′ cap requirement (Gazo et al. 2004). Its structure's lack of uninterrupted helices allows flexibility, which is crucial for its interaction with translation factors. In the case of pelargonium line pattern virus (PLPV) TED, the 5′−3′ interaction is essential for translation (Blanco-Pérez et al. 2016).

#### Barley Yellow Dwarf Virus-Like Translation Enhancer (BTE)

The BTE is predominantly found in viruses from the genera *Umbravirus*, *Dianthovirus*, *Luteovirus*, *Alphanecrovirus*, and *Betanecrovirus* of the *Tombusviridae* family (Geng et al. 2021; Ilyas et al. 2021; Shen and Miller 2004; Simon and Miller 2013; Wang et al. 2010). Structurally, BTEs exhibit a Y-shaped configuration and interact strongly with eIF4G, a key scaffolding protein in the eIF4F complex (Treder et al. 2008). Following eIF4F's binding to the BTE, the eIF4A helicase, eIF4B, and ATP bind to bring the 40S subunit straight to the BTE (Zhao et al. 2017). To scan the first AUG, the 40S complex would then be delivered to the 5′ end by the long-distance base pairing (Sharma et al. 2015). The 5′−3′ interactions and further binding of the 40S complex are mediated by eIF3 (Bhardwaj et al. 2019).

#### Panicum mosaic virus-Like Translation Enhancer (PTE)

First identified in panicum mosaic virus (PMV) and later in pea enation mosaic virus 2 (PEMV2) (Batten et al. 2006; Wang et al. 2009). Found in *Carmoviruses*, *Tombusviruses*, *Aureusvirus*-like pothos latent virus (PoLV), etc., the PTE adopts a three-way branched helical structure with a G-rich bulge in the main stem, forming a pseudoknot which is essential for recruiting eIF4E and facilitating translation (Chattopadhyay et al. 2011; Wang et al. 2009, 2011). Unlike other CITEs, the PEMV2 PTE does not establish long-distance RNA: RNA interactions with the 5′-UTR. Instead, a proximal upstream element, the kl-TSS, interacts with a 5′ hairpin within the p33 ORF, highlighting a unique translation enhancement mechanism (Gao et al. 2012). A similar CITE has also been reported from maize chlorotic mosaic virus (MCMV), referred to as MTE (Carino et al. 2020).

#### 3′-CITEs resembling T-shaped structures (TSS)

First found in TCV, resembling a 3-D tRNA-like structure consisting of two pseudoknots, three hairpins, and multiple unpaired single-stranded (ss) linker regions (McCormack et al. 2008; Zuo et al. 2010). They are primarily found in the *Carmovirus* and *Umbravirus* genera of *Tombusviridae* (Gao et al. 2012, 2013). The 80S ribosome's 60S subunit is drawn to and bound by the TCV TSS (Stupina et al. 2008). There isn't a known base pairing for this element between 3′-CITE and 5′-UTR. It was suggested that the ribosomal subunits and the UTRs create a protein bridge, with the 60S subunit binding the TSS and the 40S subunit binding the 5′-UTR (Gao et al. 2012; Stupina et al. 2008). The interaction between ribosomal subunits and TSS drives the cap-independent translation, reducing reliance on eIF4E/4G (Stupina et al. 2008).

#### 3′-CITEs resembling I-shaped structures (ISS)

The I-shaped structures (ISS) found in the 3′-UTRs of the melon necrotic spot virus (MNSV, genus *Carmovirus*) and maize necrotic spot virus (MNeSV, *Tombusvirus*) are the shortest CITEs (Miras et al. 2017a, b; Nicholson et al. 2010; Truniger et al. 2008). They appear to share a secondary structure with the TED. The I-shaped CITE can base pair with the 5′-UTR and attract ribosomes to the 5′ end of the viral fragment while bound to eIF4F (Nicholson et al. 2010). It has been demonstrated that the interacting 5′-UTR: ISS of MNeSV forms a complex in vitro with the eIF4E subunit of eIF4F (Miras et al. 2017a, b).

#### 3′-CITEs resembling Y-shaped structure (YSS)

Found in viruses belonging to the genus *Tombusvirus*, which regulates translation efficiency by long-range interaction with the 5′-UTR. Cap- and poly(A)-independent translation is facilitated by the YSS of TBSV through a 5′–3′ RNA–RNA interaction (Fabian and White 2004). YSS has also been found in pelargonium leaf curl virus (PLCV) and carnation Italian ring spot virus (CIRV), which depend on eIF4F or eIFiso4F for effective translation (Nicholson et al. 2013).

#### Cucurbit aphid-borne yellows virus (CABYV) Xinjiang-Like Translation Element (CXTE) and Mediterranean Translation Element (CMTE)

Discovered as an eIF4E resistance-breaking element from the 3′ of MNSV-N isolate. Generated due to the insertion of a 55 nt sequence from CABYV. Along with ISS, which mediates 5′–3′ RNA–RNA interaction, CXTE recruits the translation machinery and is responsible for the translation enhancement of the MNSV-N isolate (Miras et al. 2014). The 3′-CITEs of the CABYV isolates differ in the phylogenetic grouping based on geography, i.e., CXTE for the Asian, and the CMTE for the Mediterranean groups (Miras et al. 2022). The eIF4E-independent CABYV 3′-CITE activities may rely on eIF4G and PABP rather than eIF4A or the eIF4F complex (Truniger et al. 2023).

### t-RNA-like structures (TLSs)

Viruses belonging to the family *Bromoviridae,* genera *Tymovirus* and *Tobamovirus*, feature TLSs in their 3′ UTR. These TLSs mimic tRNA, contributing to viral replication and translation. They function as telomeres by interacting with nucleotidyl transferases that add a CCA tail to the 3′ end (Rao et al. 1989). They regulate replication, enhance translation, and assist in packaging viral RNA into virions (Matsuda and Dreher 2004; Annamalai and Rao 2007; Dreher 2009). The TLS in TYMV requires aminoacylation of its 3′-CCA terminus for maximum translational efficiency and works synergistically with the 5′ cap. Translation enhancement by the TLS is mediated through interactions with eEF1A, mimicking tRNA activity, and possibly involves ribosome recruitment through communication between the 3′-UTR and 5′-UTR (Matsuda et al. 2004; Colussi et al. 2015).

Conversely, in TMV, the TLS functions as a minus-strand promoter. Translation in TMV is instead driven by a 3′-UTR pseudoknot and the 5′-UTR omega (Ω) sequence, a potent cap-dependent translational enhancer recognized by heat shock protein 101 (HSP101) and eIF4F via eIF4G (Gallie and Kado 1989; Gallie and Walbot 1990; Gallie et al. 1991).

### Unique 3′ untranslated region (3′ UTR)

The AMV employs a unique mechanism to regulate RNA translation and replication via the 3′ UTR. AMV RNA requires the CP for efficient translation and infection, which interacts with the 3′-UTR, folding into a CP-binding (CPB) conformation, promoting translation by mimicking the poly(A) tail structure (Olsthoorn 1999). This CPB structure features stem-loops connected by an AUGC linker motif, critical for CP binding, potentially facilitating mRNA circularization through its interaction with translation initiation factors eIF4G/eIFiso4G (Reusken and Bol 1996; Neeleman et al. 2004; Krab et al. 2005). Furthermore, the 3′-UTR can alternate between a CPB form and a pseudoknot resembling a TLS. This pseudoknot acts as a minus-strand promoter recognized by viral replicase, suggesting that CP regulates a conformational switch between translation and replication by modulating pseudoknot stability (Chen and Olsthoorn 2010). These findings underscore the multifunctional role of AMV CP and the 3′-UTR in orchestrating viral translation and replication, highlighting a sophisticated mechanism that mimics cellular processes for efficient viral propagation.

### Genomic regions located within the coding region

Apart from the various 5′-UTRs and 3′-UTRs, the genomic regions of many viruses have been reported to interact with the eIFs to modulate various steps of the virus infection cycle. An example is the Helper Component Proteinase (HC-Pro) of *Potyviruses* and cauliflower mosaic virus (CaMV). The standard 4E-binding motif present in eIF4G is present in HC-Pro. Any mutations in PVA's HC-Pro eliminate viral infectivity (Ala-Poikela et al. 2011). VPg and HC-Pro are multifunctional potyviral proteins that interact with one another and form homodimers (Yambao et al. 2003; Roudet-Tavert et al. 2007). The HC-Pro of *Potyviruses* has also been found to interact with a gene of the eIF4E family (StnCBP), facilitating accumulation in tomato (Chen et al. 2022). CaMV utilizes a unique strategy involving its transactivator protein (TAV) to enhance translational reinitiation of viral polycistronic mRNA (Pooggin and Ryabova 2018). TAV acts as a scaffold, coordinating with initiation factors such as eIF3 and eIF4B, other host proteins, and the ribosome to facilitate this process (Thiébeauld et al. 2009). The CP of Citrus leprosis virus C (CiLV-C) interacts with eIF4A, promoting replication (Leastro et al. 2025).

## Catalogue of translation factors involved in viral susceptibility

Eukaryotic translation factors, play multifaceted roles in the virus infection cycle beyond their canonical functions in protein synthesis (Fig. 1, Table 1).

**Table 1:** Catalog of eEF and eIF genes involved in viral susceptibility

Translation factor	Virus name	Host Plant	Infection step	Reference
eEF1A	TMV	*N. benthamiana*, *N.tabacum*	Replication, Cell-to-cell movement	(Zeenko et al. 2002; Yamaji et al. 2006)
	TYMV	*Vigna unguiculata*	Translation	(Matsuda and Dreher 2004)
	BaMV	*N. benthamiana*	Translation	(Lin et al. 2007)
	TuMV	*Arabidopsis thaliana*	Replication	(Thivierge et al. 2008)
	RRSV and SRBSDV	*Oryza sativa*	Replication	(Songbai et al. 2013)
	TSWV	*N. benthamiana*	Replication, Translation, and systemic movement	(Komoda et al. 2014; Helderman et al. 2021)
	CMV and TRV	*Petunia atkinsiana*	Replication	(Sun et al. 2020)
	CWMV	*Triticum aestivum* and *N. benthamiana*	Replication and translation	(Chen et al. 2021)
eEF1A and eEF1B	SMV	*Glycine max*	Translation elongation	(Luan et al. 2016)
eEF1B	TMV	*N. benthamiana*	Replication	(Hwang et al. 2013)
	PVX	*N. benthamiana*	Replication and translation	(Hwang et al. 2015)
eIF2Bβ	TuMV	*Brassica juncea*	Translation	(Shopan et al. 2017)
eIF3	BMV	*Hordeum vulgare*	Replication	(Quadt et al. 1993)
	TMV	*Solanum lycopersicum*	Replication	(Osman and Buck 1997)
eIF3B	TuMV	*A. thaliana*	Translation	(Rubio et al. 2019a, b)
	BYDV	*H. vulgare*	Translation	(Bhardwaj et al. 2019)
eIF4A	PPV and TuMV	*Prunus persica* and *A. thaliana*	Replication and translation	(Huang et al. 2009)
	TBSV	*A. thaliana*	Replication	(Kovalev et al. 2012a, b; Kovalev and Nagy 2014)
	RSV	*N. benthamiana*, *O.sativa*	Negative regulator of anti-viral autophagy	(Zhang et al. 2021)
	CMV	*N. benthamiana*	Translation	(Li et al. 2023)
	CiLV-C	*N. benthamiana*	Replication	(Leastro et al. 2025)
eIF4E	LMV	*Lactuca sativa*	Translation, Systemic virus movement	(German-Retana et al. 2008; Nicaise et al. 2003)
	PSbMV	*Pisum sativa*	Cell-to-cell movement	(Gao et al. 2004)
	BYMV	*H. vulgare*	-	(Stein et al. 2005)
	MNSV	*A.thaliana* and *Cucumis melo*	Translation	(Nieto et al. 2006)
	TuMV	*Brassica rapa*	Replication	(Jenner et al. 2010)
	BMYV and BWYV-USA	*A.thaliana*	Translation	(Reinbold et al. 2013)
	TEV	*Capsicum annum*	Replication, Translation	(Estevan et al. 2014)
	LMV	*N.benthamiana*	Translation	(Tavert-Roudet et al. 2017)
	SCSMV	*Saccharum officinarum*	Translation	(Shan et al. 2023)
eIFiso4E	TuMV, TEV	*A.thaliana*	Translation	(Léonard et al. 2000; Lellis et al. 2002)
	CVB	*Chrysanthemum morifolium*	Translation	(Song et al. 2013)
	TEV	*A.thaliana*	Systemic virus movement	(Contreras-Paredes et al. 2013)
eIF4E and eIFiso4E	PVY	*C.annum*	Cell-to-cell movement	(Arroyo 1996; Ruffel et al. 2002)
	PVMV	*C.annum*	-	(Ruffel et al. 2006)
	SCMV	*Zea mays*	Systemic movement	(Rodríguez-Gómez et al. 2022)
*Bv-eIF(iso)4E*	PVA	*N. benthamiana*	Systemic movement	(Saha and Mäkinen 2020)
eIF4G	TCV	*A.thaliana*	Cell–cell movement	(Yoshii et al. 2004)
eIFiso4G	RYMV	*O.sativa*	Translation	(Albar et al. 2006; Boisnard et al. 2007; Hébrard et al. 2010)
	TuYV	*A. thaliana*	Translation	(Reinbold et al. 2013)
eIF4G and eIFiso4G	PVMV, RYMV	*Oryza sativa; Pisum sativum*	Translation	Ruffel et al. 2006
	ClYVV	*A.thaliana*	Translation	(Nicaise et al. 2007)
	TriMV	*T.aestivum*	Translation	(Roberts et al. 2017)
eIF4E and eIF4G	CMV	*A. thaliana*	Cell–cell movement	(Yoshii et al. 2004)
	TuMV	*N.benthamiana*	Translation	(Chen et al. 2024)
eIF(iso)4E and eIF(iso)4G	PPV, LMV, and TuMV	*A.thaliana*	Translation	(Nicaise et al. 2007)

### Eukaryotic translation elongation factors

#### Eukaryotic translation elongation factor 1 A (eEF1A)

eEF1A is a core component of the translation machinery, delivering aminoacyl-tRNA (aa-tRNA) to the ribosomal A site as part of the eEF1A-GTP-aa-tRNA ternary complex (Browning 1996; Nyborg and Liljas 1998). It catalyzes the first step of the elongation cycle, ensuring efficient protein synthesis. eEF1A is a highly abundant and evolutionarily conserved protein, encoded by multiple genes in many eukaryotes, such as rice, where at least 14 eEF1A-like genes exist (Kidou and Ejiri 1998). Beyond translation, it also plays a role in organizing the cytoskeleton and mediating nuclear-cytoplasmic trafficking (Negrutskii and El’skaya 1998; Mateyak and Kinzy 2010).

eEF1A is reported to aid in the RNA synthesis and translation of viruses from families like *Tombusviridae*, *Flaviviridae*, and *Tymoviridae*, and viruses like TMV, TuMV, soybean mosaic virus (SMV), and brome mosaic virus (BMV) (Bastin and Hall 1976; Davis et al. 2007; Li et al. 2010; Matsuda et al. 2004; Yamaji et al. 2006; Zeenko et al. 2002). eEF1A interacts with the 3′-UTR of viral RNA and the methyltransferase domain of RdRp, forming replication complexes critical for viral genome replication (Abbas et al. 2015). eEF1A is unique among negative-strand RNA viruses, assisting transcription and replication of tomato spotted wilt virus (TSWV) (Komoda et al. 2014). In the case of a DNA virus, i.e., tomato yellow leaf curl virus (TYLCV), eEF1A has been identified to inhibit replication and movement, acting as a resistance gene (Wazwaz 2013).

#### Eukaryotic translation elongation factor 1B (eEF1B)

The eEF1B is a guanine nucleotide exchange factor (GEF) that catalyzes the exchange of GDP for GTP on the eEF1A protein. This recycling is critical for the continuous function of eEF1A in translation elongation. eEF1B is a multi-subunit complex in plants, comprising a structural protein (eEF1Bγ) and two guanine nucleotide exchange subunits (eEF1Bα and eEF1Bβ) (Andersen and Nyborg 2001). eEF1Bγ has been reported to promote the (-)strand RNA synthesis of TBSV by interacting with eEF1A and a loop in the TBSV RNAs 3′ UTR (Sasvari et al. 2011). Another study demonstrated the crucial role of eEF1A and eEF1Bβ in the replication of PVX in plants, as they interact with the viral TGBp1 protein (Hwang et al. 2015). Both have also been discovered to play a crucial role in TMV replication, interacting with the 3’ cis-acting element (Yamaji et al. 2006; Hwang et al. 2013, 2015).

### Eukaryotic translation initiation factors

#### Eukaryotic translation initiation factor 2B beta (eIF2Bβ)

The eIF2Bβ is a GEF for eIF2 (GTP-binding protein crucial in translation initiation), catalyzing the exchange of GDP for GTP on eIF2, a process necessary to deliver initiator tRNA to the ribosome. The eIF2Bβ is responsible for enhanced translation of TuMV RNA in susceptible *Brassica juncea* plants. While in the case of resistant plants, the eIF2Bβ allele with a non-synonymous substitution (A120G) in its N-terminal region is encoded by a single recessive gene, retr03, that impairs TuMV infection by disrupting the virus-host interaction (Shopan et al. 2017). Recent findings suggest that eIF2Bβ provides resistance by modifying the methylation of the TuMV CP in conjunction with another interacting factor. This methylation disrupts viral replication and infection (Sha et al. 2024).

#### Eukaryotic translation Initiation Factor 3B (eIF3B)

The eIF3 is a multiprotein complex (eIF3a-m) essential for translation initiation. It stabilizes the binding of the 40S ribosomal subunit to the mRNA, promotes recruitment of the Met-tRNAi to the ribosome, and facilitates subsequent steps in the assembly of the translation initiation complex. eIF3 has been reported to interact with the 2a protein of BMV, i.e., RdRp, indicating a role in replication (Quadt et al. 1993). eIF3-mediated viral protein synthesis has been observed in the case of CaMV, wherein an eIF3–TAV–RISP–L24 complex is formed inside the host cell, as discussed earlier (Thiébeauld et al. 2009). According to recent research on the BYDV, eIF3 binds to both 5′ and 3′ UTRs (BTE) at the same time, hence promoting communication between both initiating BYDV translation (Powell et al. 2022). The eIF3b is a component of RTM3, a dominant resistant allele against TuMV (Rubio et al. 2019a, b).

#### Eukaryotic translation Initiation Factor 4 A (eIF4A)

The eIF4A is a highly conserved DEAD-box RNA helicase that plays a pivotal role in the initiation of translation in plants (Rogers et al. 2002). As a component of the eIF4F complex, eIF4A is primarily responsible for unwinding of secondary structures in the 5′ UTRs of mRNAs (Parsyan et al. 2011). This unwinding facilitates the scanning and binding of the small ribosomal subunit to locate the start codon, a critical step in cap-dependent translation initiation. The role of eIF4A has been implicated in the replication and translation of many animal and human viruses (Taroncher-Oldenburg et al. 2021). However, fewer studies have been conducted on plant-infecting viruses. Studies carried out in the yeast system indicated the mediation of translation of RNA2 of BMV and replication of the (-) strand of TBSV via eIF4A (Kovalev et al. 2012a, b; Noueiry et al. 2000). Another study reported similar helicases in peach and Arabidopsis binding to VPg of *Potyviruses* (plum pox virus (PPV) and TuMV), enhancing replication and translation (Li et al. 2016). Similar helicases in *A.thaliana* have been implicated in aiding replication of TBSV (Kovalev et al. 2012a; Kovalev and Nagy 2014). Recently, the essential role of eIF4A in the translation of cucumber mosaic virus (CMV) RNA and replication of CiLV-C has been demonstrated (Leastro et al. 2025; Li et al. 2023). eIF4A also acts as a negative regulator of antiviral autophagy in both hosts by interacting with the Autophagy-related genes (ATGs) (Zhang et al. 2021).

#### Eukaryotic translation Initiation Factor 4B (eIF4E)

The eIF4E plays a pivotal role in the initiation of translation by binding to the 5' cap structure of mRNA, facilitating the recruitment of the ribosome. Plant viruses, particularly belonging to the potyvirus group, exploit the cap-binding ability of eIF4E to facilitate their replication, translation, and movement within the host plant (Zlobin and Taranov 2023). As mentioned earlier, viruses possess a particular protein, i.e., VPg, that competes with host mRNAs for binding to eIF4E/eIFiso4E, enhancing viral genome translation (Léonard et al. 2000). Specific amino acid substitutions in the cap-binding pocket of eIF4E and its isoforms were found to be critical for interaction with virus VPg. In case of viruses lacking VPg, structures such as 3′ CITEs lead to 5′−3′ interaction, facilitating interaction with eIF4E/eIFiso4E for translation (Sanfaçon 2015).

The eIF4E/eIFiso4E also play role in the intercellular movement of viruses, as demonstrated for pepper and pea plants that exhibit recessive resistance to PVY and pea seed-borne mosaic virus (PSbMV), respectively, due to mutations in the eIF4E gene, which can be linked to faults in the virus's cell-to-cell movement (Arroyo 1996; Gao et al. 2004). The long-distance movement of lettuce mosaic virus (LMV) has been observed to be hampered by the eIF4E-mediated resistance in lettuce (German-Retana et al. 2008). The eIF4E in maize has also been attributed to influence long-distance movement of sugarcane mosaic virus (SCMV) (Rodríguez-Gómez et al. 2022). In addition, studies have shown that a deficiency in the eIF4E isoform nCBP limits the cell-to-cell movement of a potexvirus in *A.thaliana*, highlighting its importance in the systemic spread of the virus within the host (Keima et al. 2017).

#### Eukaryotic translation Initiation Factor 4G (eIF4G)

The eIF4G serves as a scaffold protein that orchestrates the assembly of various initiation factors necessary to recruit ribosomes to mRNA. As discussed, eIF4G has been reported to mediate the translation of plant viral RNAs, facilitating infection that lacks the conventional 5' cap structure necessary for ribosome binding. As seen in the case of RYMV, wherein eIF(iso)4G interacts with the VPg, initiating translation (Albar et al. 2006; Hébrard et al. 2010). Similarly, eIF4G binds to the conserved stem loop (BTE) of BYDV, 3′-CITE of CABYV, and IRES of TriMV, facilitating translation initiation (Kraft et al. 2013; Roberts et al. 2017).

#### Eukaryotic translation Initiation Factor 5 A (eIF5A)

The eIF5A is now understood to facilitate the elongation phase of protein synthesis by resolving ribosomal stalling, particularly at sequences rich in proline residues. This ensures the efficient synthesis of proline-rich proteins. Given eIF5A's role in facilitating translation elongation, it is plausible that viruses exploit this factor to enhance the synthesis of viral proteins, thereby promoting replication and systemic movement within the host plant. Some studies suggest that eIF5A might regulate programmed cell death (PCD) pathways, which are crucial for limiting viral spread. Mutations affecting eIF5A function could alter PCD responses, influencing the outcome of viral infections (Hopkins et al. 2008).

#### Eukaryotic translation Initiation Factor 6 (eIF6)

The eIF6 plays a pivotal role in ribosome biogenesis. It binds to the 60S ribosomal subunit, preventing its premature association with the 40S subunit, thereby regulating the formation of functional 80S ribosomes essential for protein synthesis (Guo et al. 2011). Beyond its fundamental role in translation, eIF6 has been implicated in plant-virus interactions. For example, the nuclear inclusion protein b (NIb) of TuMV has been shown to weaken the function of eIF6, thereby promoting viral infection in *Nicotiana benthamiana* (Chen et al. 2024). This interaction suggests that viruses may exploit eIF6 to enhance their replication and systemic movement within the host plant. It is a potential susceptibility factor that certain viruses can manipulate to establish successful infections.

## Strategies to develop virus resistance using eIF knowledge

**Fig. 2:**
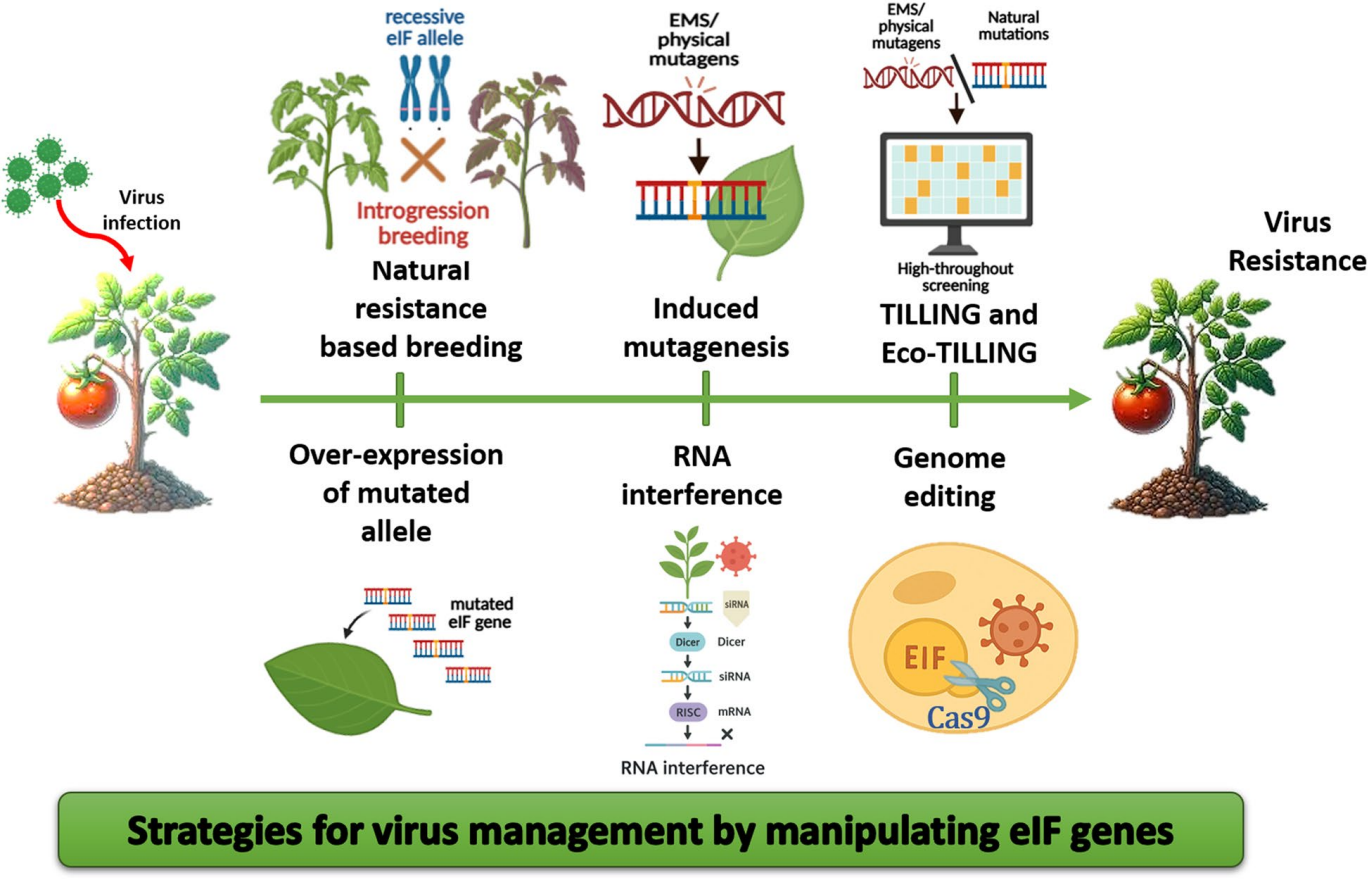
Schematic representation of the various strategies that are in use for the manipulation of eukaryotic translation factors for management

The viral resistance based on eTFs occurs through loss of susceptibility in hosts, and is considered more durable and widespread (Choudhary and Suresh 2020; Truniger and Aranda 2009). The resistance conferred by mutant eTFs is often due to disrupting the interaction between the eTFs and viral proteins. This interference prevents the virus from hijacking the host's translation machinery, inhibiting viral replication, translation, and movement. The recessive resistance due to natural or induced mutations is usually due to a single nucleotide change in the eIF gene, which in turn leads to bypassing of the interacting viral region, as observed in the case of VPg-eIF interaction (Duprat et al. 2002). Since this kind of resistance is based on the non-availability of an essential factor in the host for virus proliferation, it is inherited recessively. As a single host, eEF or eIF may mediate the infection cycle of many viruses; this approach can be used to induce resistance against multiple viruses at once (Diaz‐Pendon et al. 2004). It can be either naturally present in hosts or induced artificially via various approaches, as discussed below and represented in Fig. 2.

### Natural resistance-based breeding

In classical and molecular breeding efforts, allele mining of eIFs' natural genetic variation has emerged as a key tactic. Natural mutations have been found in many eIF genes that impair interaction with viral proteins, often leading to recessive resistance, where the loss or alteration of the host susceptibility factor prevents the virus from completing its life cycle without invoking a defence response. Mutant forms of translation initiation factors, such as eIF2Bβ, eIF4E, eIF4G, and their isoforms, eIF(iso)4E and eIF(iso)4G, are frequently encoded by these recessive resistance genes (Table 2). For example, in *S. habrochaites* (a tomato wild cousin), the allele Sh–eIF4E1^PI24^–pot1 has been found to mediate resistance to numerous strains of TEV and PYV (Ruffel et al. 2005). Such resistance is conferred by point mutation, as seen in the case of resistance to zucchini yellow mosaic virus (ZYMV) and papaya ringspot virus-W (PRSV-W) in watermelon, conferred by the mutation D71G in eIF4E. In contrast, resistance to PVY was conferred by the corresponding mutation D55G in tobacco eIF4E (Zhou et al. 2024a). Activation of the GCN2–eIF2α module is another antiviral defense strategy by restricting synthesis of viral proteins and hampering replication, as has been widely documented in non-plant systems. However, its potential use in plant-virus system needs to be researched. Interestingly, natural recessive resistance based on loss-of-function alleles in eIFs is widely used and is often considered durable since it reduces direct selection pressure on viral avirulence genes. However few studies of resistance breakdown have been reported such as, mutations in the potyviral VPg can shift interaction specificity from eIF4E to eIF(iso)4E, thereby overcoming eIF4E-mediated resistance (Charron et al. 2008; Moury et al. 2014). Also, since viruses cannot easily bypass the need for specific host factors without compromising their life cycle, evolutionary constraints limit the emergence of resistance-breaking strains (Pavan et al. 2010; Truniger and Aranda 2009). Breeding approaches such as classical and marker-assisted selection (MAS) have been widely employed to introgress these natural recessive alleles into elite backgrounds. For example, the rym4 and rym5 alleles have been successfully introgressed into barley breeding lines across Europe and Asia to control barley yellow mosaic virus/barley mild mosaic virus (BaYMV/BaMMV) (Perovic et al. 2014). Similarly, resistant lines of tomato, lettuce, and melon have been developed using pot1, mo1, and nsv alleles, respectively (Robaglia and Caranta 2006). A significant benefit of classical breeding is its widespread public acceptance, as it does not involve transgenic modification and pyramiding, enabling the stacking of multiple resistance genes. However, the process is time-consuming and labour-intensive, and often carries the risk of retaining unwanted traits from donor plants. Moreover, eliminating undesirable genetic linkages can be difficult, especially when dealing with traits governed by multiple loci.

**Table 2:** List of translation factor modifications employed to manage plant viruses

Virus name	Host Plant	Translation factor	Gene/construct	Reference
Natural Recessive Resistance				
PVY, TEV	Pepper	eIF4E	pvr1	(Kang et al. 2005; Ruffel et al. 2002)
LMV	Lettuce	eIF4E	mo1	(Nicaise et al. 2003)
PVY, TEV	Tomato	eIF4E	pot1	(Ruffel et al. 2005)
BaMMV, BaYMV	Barley	eIF4E	rym	(Kanyuka et al. 2005)
BaMMV, BaYMV	Barley	eIF4E	rym5	(Stein et al. 2005)
RYMV	Rice	eIF(iso)4G	Rymv1	(Albar et al. 2006)
TuMV	Chinese cabbage	eIFiso4E	retr01, retr02	(Rusholme et al. 2007)
PsBMV, BYMV	Pea	eIF4E	sbm-1	(Bruun-Rasmussen et al. 2007)
ClYVV	Pea	eIF4E	cyv-2	(Andrade et al. 2009)
PPV	Apricot	eIF4E	QTLs	(Marandel et al. 2009)
ZYMV	Watermelon	eIF4E	SNP	(Ling et al. 2009)
PVMV, ChiVMV	Pepper	eIF4E and eIF(iso)4E	pvr2 and pvr6	(Hwang et al. 2009; Rubio et al. 2009; Ruffel et al. 2002)
RTSV	Rice	eIF4G	tsv1	(Lee et al. 2010)
BCMV, ClYVV	Common bean	eIF4E	bc-3	(Naderpour et al. 2010; Hart and Griffiths 2013)
PsBMV, BYMV	Pea	eIF4E	sbm-1	(Ashby et al. 2011)
TuMV	Chinese cabbage	eIFiso4E	retr01, retr02	(Qian et al. 2013)
BaMMV, BaYMV	Barley	eIF4E	rym/rym5	(Perovic et al. 2014)
TuMV	Chinese cabbage	eIFiso4E	retr01, retr02	(Nellist et al. 2014)
TVMV	Tobacco	eIF4E	va	(Julio et al. 2015)
TuMV	Indian Mustard	eIF2Bβ	retr03	(Shopan et al. 2017)
TuMV	*A.thaliana*	eIF3b	RTM3	(Rubio et al. 2019a, b)
BaYMV and BaMMV	*Barley*	eIF4E	eIF4E_HOR3298_ allele	(Shi et al. 2019)
PVY	Tobacco	eIF4E	va	(Lin et al. 2021)
PPV	*Prunus* sp.	eIF(iso)4E	—	(Tricon et al. 2023)
Induced Mutations				
TuMV	*A. thaliana*	eIF(iso)4E	EMS	(Lellis et al. 2002)
ClYVV, TuMV	*A. thaliana*	eIF4E or or eIF(iso)4E	EMS	(Sato et al. 2005)
PVMV	Pepper	eIF4E or eIF(iso)4E	-	(Ruffel et al. 2006)
PVY	Tobacco	eIF4E1-S and eIF(iso)4E-T	EMS	(Udagawa et al. 2021)
TILLING				
MNSV	Melon	eIF4E	Eco-TILLING	(Nieto et al. 2007)
Potyviruses	Tomato	eIF4E	TILLING and Eco-TILLING	(Rigola et al. 2009)
PVY and PepMoV	Tomato	eIF4E	TILLING	(Piron et al. 2010)
PVY	Capsicum	eIF4E and eIF(iso)4E	Eco-TILLING	(Ibiza et al. 2010)
TEV	Capsicum	eIF4E	HRM	(Jeong et al. 2012)
Potyviruses	*S. habrochaites*	eIF4E	TILLING	(Gauffier et al. 2016)
PVY	Tobacco	eIF4E	TILLING	(Zhao et al. 2019)
PVMV	Tomato	eIF4E1	TILLING	(Moury et al. 2020)
Over-expression of the mutated allele				
TEV, PepMoV	Tomato	eIF4E	Overexpression of the pvr1 allele from Capsicum	(Kang et al. 2007)
PVY	Potato	eIF4E	Overexpression of the pvr2 allele from Capsicum	(Cavatorta et al. 2011)
PVY	Potato	eIF4E	Overexpression of wild potato eIF4E-1 variant Eva1	(Duan et al. 2012)
TuMV	*Brassica rapa*	eIF(iso)4E	Mutant forms (W95L, K150E)	(Kim et al. 2014)
PVY	Potato	eIF4E	pvr1^2^	(Gutierrez Sanchez et al. 2020)
RNAi				
Many potyviruses	Tomato	eIF4E-1 and eIF4E-1	hpRNA	(Mazier et al. 2011)
TBSV	*N. benthamiana*	eEF1Bγ	VIGS	(Sasvari et al. 2011)
CVYV, MNSV, MWMV, ZYMV	Melon	eIF4E	hpRNA	(Rodríguez‐Hernández et al. 2012)
PPV	Plum	eIF(iso)4E	hpRNA	(Wang et al. 2013)
TMV	*N.benthamiana* and pepper	eEF1β	VIGS	(Hwang et al. 2013)
PStV	Peanut	PeaeIF4E and PeaeIF(iso)4E	VIGS vector	(Xu et al. 2017)
PVY	Tobacco	eIFiso4E	hpRNA	(Takakura et al. 2018)
PPV	Prunus salicina	eIFiso4G	hpRNA	(Rubio et al. 2019a, b)
WSMV, TriMV	Wheat	eIF(iso)4E-2 and eIF4G	Hairpin construct	(Rupp et al. 2019)
SMV, BCMV, WMV	Soybean	eIF4E	RNAi construct	(Gao et al. 2020)
PVY	Potato	eIF4E	hpRNA	(Miroshnichenko et al. 2020)
PPV	Sour cherry	eIFiso4E	hpRNA	(Mourenets et al. 2022)
Genome editing				
TuMV	*A.thaliana*	eIF(iso)4E	sgRNA	(Pyott et al. 2016)
CVYV, ZYMV and PRSV-W	Cucumber	eIF4E	sgRNA	(Chandrasekaran et al. 2016)
RTSV	Rice	eIF4G	sgRNA	(Macovei et al. 2018)
CBSV	Cassava	eIF4E isoforms (nCBP-1 and nCBP-2)	sgRNA	(Gomez et al. 2019)
ClYVV	Arabidopsis	eIF4E	sgRNA	(Bastet et al. 2019)
PepMoV	Tomato	eIF4E1	sgRNA	(Yoon et al. 2020)
PVY and CMV	Tomato	eIF4E1	gRNA	(Atarashi et al. 2020)
WSSMV and WYMV	Wheat	eIF4E and eIF(iso)4E	sgRNA	(Hahn et al. 2021)
RBSDV	Rice	eIF4G	gRNA	(Wang et al. 2021)
BaMMV	Winter barley	eIF4E	gRNA	(Hoffie et al. 2021)
PVY	Tomato	SleIF4E1; SleIF4E2	sgRNA	(Kumar et al. 2022)
PVY	Tobacco	eIF4E1 and eIF4E2	Dual gRNA	(Le et al. 2022)
PVY	Potato	eIF4E	sgRNA	(Lucioli et al. 2022; Noureen et al. 2022)
MWMV	Melon	eIF4E	gRNA	(Pechar et al. 2022)
Multiple potyviruses	Cherry Tomato	eIF4E1	TALEN	(Kuroiwa et al. 2023)
WMV, ZYMV, and PRSV	Cucumber	eIF4E	gRNA	(Fidan et al. 2023)
TuMV	*Brassica* sp.	eIF(iso)4E	gRNA	(Lee et al. 2023)
WYMV	Wheat	eIF4E	sgRNA	(Kan et al. 2023)
Tomato viruses	Tomato	eIF1B	sgRNA	(Baranov et al. 2023)
ZYMV	Watermelon	eIF4E	sgRNA	(Li et al. 2024)
MLN	Maize	eIF4E	sgRNA	(Wen et al. 2024)
BChV	sugar beet	eIF(iso)4E	gRNA	(Rollwage et al. 2024)
TuMV	*Brassica rapa*	eIF(iso)4E.c	gRNA	(Liu et al. 2024)
TEV	*N. benthamiana*	eEF1Bγ	VIGE	(Kang et al. 2025)
PVY, TVBMV and ChiVMV	Tobacco	eIF4E and isoform	gRNA	(Liu et al. 2025)

### Induced mutations

While natural resistance alleles in eIF genes have been instrumental in breeding virus-resistant varieties, such alleles are not always available in all crop species or for all viral pathosystems. To address this limitation, induced mutagenesis through chemical or physical mutagens offers a powerful strategy to generate novel, recessive resistance alleles by artificially creating mutations in target genes. The mutated versions of eIF genes or their isoforms in plants display resistance features against viral pathogens, hindering the functional plant virus interactions. For example, EMS-induced mutations in eIF4E or eIF(iso)4E in a range of plant species have been shown to confer resistance to some *Potyviruses* (Lellis et al. 2002; Sato et al. 2005). This approach offers advantages, including non-transgenic status, broad crop applicability, and direct compatibility with breeding programs. However, there are certain limitations, such as potential pleiotropic effects, labour-intensive screening, and the possibility of viral adaptation overcoming resistance.

### TILLING (targeting induced local lesions in genomes) and Eco-TILLING (screening of targeted natural variation)

TILLING (Targeting Induced Local Lesions in Genomes) is a reverse genetics technique that combines chemical mutagenesis to induce point mutations with high-throughput screening to identify those point mutations in specific genes (McCallum et al. 2000). It enables the detection of DNA polymorphisms such as single nucleotide polymorphisms (SNPs), small insertions and deletions (InDels), haplotypes at specific genes, and variations in microsatellite (SSR) repeat numbers without involving transgenic methods (Henikoff et al. 2004). A prime example is the eIF4E1 mutation in tomato, identified through EMS mutagenesis and subsequent TILLING analysis. This mutation, located in the splice site of intron 1, resulted in a truncated, non-functional eIF4E protein that conferred complete resistance to PVY and pepper mottle virus (PepMoV) (Piron et al. 2010). Notably, the mutation had no adverse effects on plant development due to functional redundancy with eIF4E2. In tobacco, X-ray-induced mutagenesis was employed to develop the recessive resistance trait, which was later found to be associated with a gene encoding the eIF4E (Julio et al. 2015). Eco-TILLING applies the same screening principle to natural populations rather than mutagenized ones. It detects naturally occurring polymorphisms in key genes, helping uncover alleles linked to disease resistance, abiotic stress tolerance, and improved nutritional quality (Comai et al. 2004). In *Cucumis* spp., Eco-TILLING revealed eIF4E allelic variants associated with resistance to MNSV, with single amino acid substitutions located outside the canonical cap-binding site but still affecting the protein’s ability to interact with viral RNA elements (Nieto et al. 2007). This approach has been used widely to identify allelic variants and to manage plant viruses (Table 2).

TILLING and Eco-TILLING are non-transgenic methods ideal for identifying mutations in target genes, especially in regions with GMO restrictions. They enable high-throughput screening of large populations without prior knowledge of virus-host interactions and often replicate naturally occurring recessive resistance, making them suitable for conventional breeding. For instance, the tomato eIF4E1 mutant has been backcrossed into commercial cultivars to develop PVY-resistant lines (Piron et al. 2010). However, it is a time and labour-consuming technique, and impedes gene function analysis. The limitations of TILLING and Eco-TILLING are overcome by a new technique developed recently, i.e., high-resolution melting (HRM) analysis (Wittwer et al. 2003). HRM is a highly effective and sensitive technique for identifying sequence changes in plants, such as SNPs, insertions, and deletions (InDels), and even microsatellites (Jeong et al. 2012). This approach has been utilized to identify variations in eIF4E, leading to TEV-HAT strain resistance in capsicum (Jeong et al. 2012).

Advantages include generation of an allelic series (which allows fine-tuning gene function) and relative ease of moving promising alleles into elite backgrounds, but limitations are that mutations are random (necessitating extensive screening), can create linked undesirable changes (requiring backcrossing), and it can be slower than targeted editing for specific hypotheses (Szurman-Zubrzycka et al. 2023). TILLING has contributed practical, field-tested variants in cereals and other crops and remains widely used for pre-breeding and allele mining. To reduce resistance breakdown, rational application of TILLING involves isolating multiple independent alleles of key susceptibility genes (to avoid single-allele vulnerabilities), pyramiding favorable alleles by breeding, and combining induced alleles with other resistance mechanisms discovered by genomics.

### Over-expression of the mutated susceptible allele

Transgenic overexpression of altered, non-functional, or competitive eIF alleles that prevent viral infection without affecting regular plant translation is another method of inducing resistance. This method involves introducing a mutant form of an eIF gene under a constitutive or inducible promoter in a susceptible plant background. Usually, the mutant form of the gene hinders viral contact (e.g., impaired VPg binding). By successfully sequestering the viral protein (such as VPg) and impeding viral RNA translation or replication, the mutant eIF protein may compete with the normal, standard, functioning form. In susceptible cultivars of *Brassica rapa*, overexpression of eIF(iso)4E mutants (W95L and K150E) provided broad-spectrum resistance against several isolates of the TuMV, inhibiting interaction with VPg (Kim et al. 2014). Similarly, in potatoes, overexpression of a modified eIF4E regulated resistance to PVY (Gutierrez Sanchez et al. 2020). On pairing with other resistance genes, this strategy can confer dominant, broad-spectrum viral resistance even in genetically vulnerable individuals. Being a transgenic strategy, it can encounter acceptance and regulatory obstacles, eventually resulting in unexpected effects or issues in virus adaptability.

### RNA silencing

RNA interference (RNAi) has emerged as a prevailing strategy to engineer plant virus resistance by silencing host susceptibility factors. Unlike conventional transgenic approaches that express resistance genes or modified alleles, RNAi offers post-transcriptional gene silencing (PTGS) of specific host genes, thereby blocking viral replication and movement. RNAi works via the production of double-stranded RNA (dsRNA) homologous to the target gene that is processed by Dicer-like (DCL) enzymes into small interfering RNAs (siRNAs), triggering the gene silencing pathway, activating RNA-induced silencing complex (RISC). The RISC then degrades or inhibits the translation of complementary mRNAs, thus breaking the virus infection cycle (Viswanath Kasi et al. 2023). This strategy effectively mimics recessive resistance by functionally knocking down the expression of key host factors, often with greater flexibility and tunability.

Knocking down susceptibility factors like eIF4E and other eIF genes through RNAi has proven effective in generating virus resistance in crops such as tomato, cucumber, plum, etc. (Table 2). This method also works in polyploid plants, as shown in tobacco, where silencing both eIF(iso)4E homeologs reduced infection by a resistance-breaking PVY strain (Takakura et al. 2018). In recent years, there has been a promising surge in developing efficient and effective methods for delivering dsRNA to induce RNAi. Spray-induced gene silencing (SIGS) has been proven to be an effective delivery strategy for dsRNA against virus infection in plants (Das and Sherif 2020). One primary concern is the possibility of off-target effects, where genes other than the intended targets are inadvertently silenced due to sequence similarities. This can lead to unexpected and sometimes harmful changes in the plant (Gandhi et al. 2021). Other limitations include variable systemic RNA delivery for topical approaches, stability of applied dsRNA in the field, and regulatory/public acceptance of transgenic approaches in some regions. Also, field-level performance remains inconsistent, as resistance observed under controlled laboratory conditions can be compromised by environmental variability and diverse viral populations. While RNAi-based strategies show potential for systemic protection, their longevity and stability under open-field conditions remain a challenge (Barroso et al. 2025).

Encouragingly, exogenous delivery platforms (e.g., BioClay formulations) and optimized dsRNA designs have shown protective effects under field or semi-field conditions, demonstrating a non-transgenic route to practical use (Halder et al. 2022). To lower resistance breakdown risk, rational strategies include designing dsRNAs that target multiple conserved viral genes or host factors, combining transgenic RNAi with cultural or genetic resistance, and monitoring virus sequence diversity to update target sequences.

### Genome editing

The emergence of genome editing has revolutionized the development of virus-resistant crops lacking natural resistance alleles by enabling precise, targeted modifications of host susceptibility genes via DNA repair mechanisms activated by site-specific endonucleases, such as zinc finger nucleases (ZFNs), transcription activator-like effector nucleases (TALENs), and CRISPR-Cas (Clustered Regularly Interspaced Short Palindromic Repeats and CRISPR-associated proteins) (Azeez et al. 2024). The CRISPR-Cas system involves an RNA-guided endonuclease for cleavage and a Protospacer adjacent motif (PAM) for specificity (Makarova and Koonin 2015). Class II CRISPR/Cas systems are extensively used as potential gene editing methods to manage plant viral infections. They speed up plant protection because of their high target accuracy, ease of use, efficiency, and multiplexing capabilities. CRISPRi is another powerful addition to the CRISPR toolkit, enabling precise gene silencing without altering the DNA sequence (Ghavami and Pandi 2021). Virus-induced gene editing (VIGE) is an updated approach that produces gene-edited plants without tissue culture by delivering single-guide RNAs (sgRNAs) into Cas9-overexpressing plants using viral vectors (Ali et al. 2015; Oh et al. 2021).

CRISPR-Cas technology has become a promising tool for precise genome editing of these susceptibility genes to inhibit virus replication (Bastet et al. 2017) (Table 2). This approach offers advantages such as high precision, versatility, non-transgenic compatibility with multiple crop genomes, and most importantly, multiplexing, wherein two or more genes can be edited simultaneously to enhance durability (Kumar et al. 2022). CRISPR-based strategies, though powerful in laboratory and greenhouse settings, still face barriers related to regulatory acceptance, off-target risks, somaclonal variation from tissue culture and resistance durability in the field (Ajayi et al. 2024). Rational design approaches, including multiplex editing or allele stacking, use of base editors to make subtle loss-of-function alleles rather than complete knockouts (reducing fitness costs), and combining edits with conventional resistance loci for durability (Zhan et al. 2024).

## Conclusion and future perspectives

Plant translation is a highly regulated process that is largely coordinated by eukaryotic translation factors (eEFs and eIFs). In addition to their natural roles in protein synthesis, these elements have become important host susceptibility factors that plant viruses use to carry out their infection cycles, which include translation, replication, and cell-to-cell spread. The complex interaction between viral proteins and plant eEFs and eIFs reveals a molecular battlefield that determines the resistance or success of an infection. Understanding these interactions provides insights into viral pathogenicity and potential avenues for developing resistance strategies. The eEFs and eIFs have been characterised in various crops, mediating the virus infection cycle. However, there is a pressing need to functionally characterize the full complement of eEFs and eIFs genes across a broader range of crops using CRISPR/Cas-based knockouts, transcriptomic profiling, and protein–protein interaction studies. As viruses co-evolve rapidly, studying the structural flexibility and mutational adaptability of viral proteins (such as VPg or IRES elements) that interact with eIFs will be crucial to predicting and mitigating resistance breakdown. High-throughput phenotyping combined with allele mining through pan-genomics and genome-wide association studies (GWAS) will make it easier to find rare, potent eIF resistance alleles that can be used in genomic or marker-assisted selection.

Viruses in isolation are uncommon in field environments; instead, crops are subjected to a variety of pathogens and abiotic stresses at the same time. In cellular stress responses, translation factors themselves serve as convergence sites. For example, some eIF4E and eIF5A alleles have been connected to both abiotic stress tolerance and viral susceptibility. Plant performance may be inadvertently compromised if eIF alleles are engineered without taking these cross-stress trade-offs into account. Therefore, future strategies must take an integrated approach, stacking eIF variations with other resilience genes and developing resistance alleles that maintain translational efficiency under heat or drought stress. Recent reviews emphasize the potential of single-cell omics, ribosome profiling (Ribo-seq), and emerging “translatomics” approaches to capture cell-to-cell heterogeneity in translational responses during infection (Samarskaya et al. 2025). Such integrative omics approaches have already proven transformative in mammalian virology and are now being extended to plant systems, promising to reveal how potyviruses selectively reprogram host translation to favor viral protein synthesis. Collectively, the convergence of structural biology with multi-omics, translatomics, and pangenomics offers an unprecedented opportunity to move beyond descriptive studies of virus genome–eIF binding toward a holistic and mechanistic understanding of viral pathogenesis and durable resistance strategies.

Diverse resistance strategies—ranging from natural allele mining and induced mutagenesis to advanced genome editing and RNA interference—have been effectively employed to manipulate eIF genes and disrupt virus-host compatibility. eIF-based virus resistance fits naturally within “green control” paradigms, which emphasize reduced chemical inputs and environmentally sustainable plant protection. Importantly, these approaches offer alternatives to conventional R gene-mediated resistance, often providing broader-spectrum and more durable resistance due to lower selective pressure on viral avirulence genes. However, mutations in the eIF-interacting virus gene enable viruses to overcome recessive resistance genes in hosts (Takakura et al. 2018). This was observed recently in watermelon, wherein natural variation in the α1-α2 loop of VPg enables it to interact with eIF(iso)4E, breaking eIF4E-mediated resistance. Therefore, combining eIF-based recessive resistance with other layers of defense—such as RNAi, R genes, or antiviral peptides—could offer robust and broad-spectrum protection, particularly against mixed infections and emerging viral strains.

In conclusion, leveraging our growing understanding of eIF biology opens up an exciting frontier in plant virology and crop improvement. With strategic integration of molecular, breeding, and biotechnological approaches, the goal of durable, broad-spectrum viral resistance through targeted manipulation of translation initiation factors is becoming an achievable reality.

## Data Availability

Not applicable.

## Abbreviations

AMV: Alfalfa mosaic virus
BaMMV: Barley mild mosaic virus
BYDV: Barley yellow dwarf virus
BaYMV: Barley yellow mosaic virus
BCMV: Bean common mosaic virus
BYMV: Bean yellow mosaic virus
BWYV-USA: Beet western yellows virus-USA
BChV: Beet chlorosis virus
BMYV: Beet mild yellowing virus
CBSV: Cassava brown streak virus
CVB: Chrysanthemum virus B
CMV: Cucumber mosaic virus
ClYVV: Clover yellow vein virus
CWMV: Chinese wheat mosaic virus
ChiVMV: Chilli veinal mottle virus
CVYV: Cucumber vein yellowing virus
LMV: Lettuce Mosaic Virus
MLN: Maize lethal necrosis
MCMV: Maize chlorotic mosaic virus
MNSV: Melon necrotic spot virus
MWMV: Moroccan watermelon mosaic virus
BMV: Brome mosaic virus
PRSV-W: Papaya ring spot mosaic virus* ‐W*
PsBMV: Pea seed-borne mosaic
PLPV: Pelargonium line pattern virus
PStV: Peanut stripe virus
PPV: Plum pox virus
PepMoV: Pepper mottle virus
PVY: Potato virus Y
PVX: Potato virus X
RBSDV: Rice black-streaked dwarf virus
RRSV: Rice Ragged Stunt Virus
RSV: Rice stripe virus
RTM3: Restricted TEV movement 3
RTSV: Rice tungro spherical virus
RYMV: Rice yellow mottle virus
SCSMV: Sugarcane streak mosaic virus
SMV: Soybean mosaic virus
SRBSDV: Southern Rice Black-Streaked Dwarf Virus
TriMV: Triticum mosaic virus
TCV: Turnip crinkle virus
TEV: Tobacco Etch Virus
TMV: Tobacco mosaic virus
TRV: Tobacco rattle virus
TuMV: Turnip Mosaic Virus
TVMV: Tobacco vein mottling virus
TVBMV: Tobacco vein banding mosaic virus
TBSV: Tomato bushy stunt virus
TYMV: Turnip yellow mosaic virus
TYLCCNV: Tomato yellow leaf curl China virus
UTR: Untranslated region
WSSMV: Wheat spindle streak mosaic virus
WYMV: Wheat yellow mosaic virus
WMV: Watermelon mosaic virus
WSMV: Wheat streak mosaic virus
ZYMV: Zucchini yellow mosaic virus
